# Supplementary material for: FERN – a Java framework for stochastic simulation and evaluation of reaction networks
Source: BMC Bioinformatics. 2008 Aug 29;9:356. doi: 10.1186/1471-2105-9-356 (PMC2553347; doi:10.1186/1471-2105-9-356)
Supplement: Additional file 1 — FERN distribution, Version 1.3. This archive contains the FERN source code and binaries as well as documentation and example models in FernML and SBML. [file 1471-2105-9-356-S1.zip › fern/doc/javadoc/fern/network/sbml/class-use/MathTree.Node.html]

Uses of Class fern.network.sbml.MathTree.Node


---


|  |  |  |
| --- | --- | --- |
| **Overview** \| **Package** \| **Class** \| **Use** \| **Tree** \| **Deprecated** \| **Index** \| **Help** | |  |
| PREV   NEXT | **FRAMES**    **NO FRAMES**     **All Classes** |  |


---


## **Uses of Class fern.network.sbml.MathTree.Node**

| Packages that use MathTree.Node | |
| --- | --- |
| **fern.network.sbml** | Provides the classes for parsing and using sbml based networks. |

Uses of MathTree.Node in fern.network.sbml

| Subclasses of MathTree.Node in fern.network.sbml | |
| --- | --- |
| `static class` | `MathTree.ConstLeaf` |
| `static class` | `MathTree.GlobalLeaf` |
| `static class` | `MathTree.InnerNode` |
| `static class` | `MathTree.VarLeaf` |

| Fields in fern.network.sbml declared as MathTree.Node | |
| --- | --- |
| `MathTree.Node[]` | `MathTree.InnerNode.Children` |

| Methods in fern.network.sbml that return MathTree.Node | |
| --- | --- |
| `MathTree.Node` | `MathTree.getRoot()`             Gets the root of this MathTree. |

Constructors in fern.network.sbml with parameters of type MathTree.Node

`MathTree.InnerNode(MathTree.Node[] Children,
int AstNodeType)`

---


|  |  |  |
| --- | --- | --- |
| **Overview** \| **Package** \| **Class** \| **Use** \| **Tree** \| **Deprecated** \| **Index** \| **Help** | |  |
| PREV   NEXT | **FRAMES**    **NO FRAMES**     **All Classes** |  |


---
